# Multi-Micro/Nanolayer Films Based on Polyolefins: New Approaches from Eco-Design to Recycling

**DOI:** 10.3390/polym13030413

**Published:** 2021-01-28

**Authors:** Geraldine Cabrera, Ibtissam Touil, Emna Masghouni, Abderrahim Maazouz, Khalid Lamnawar

**Affiliations:** 1 Ingénierie des Matériaux Polymères, UMR 5223 INSA Lyon, Université de Lyon, CNRS, F-69621 Villeurbanne, France; geralcabrera09@gmail.com (G.C.); Ibtissam.touil@insa-lyon.fr (I.T.); emna.masghouni@insa-lyon.fr (E.M.); abderrahim.maazouz@insa-lyon.fr (A.M.); 2 Hassan II Academy of Science and Technology, Rabat 10100, Morocco; 3 Fujian Key Laboratory of Polymer Science, Fujian Normal University, Fuzhou 350007, China

**Keywords:** recycling, eco-design, coextrusion, multilayers

## Abstract

This paper describes a future-oriented approach for the valorization of polyethylene-based multilayer films. The method involves going from eco-design to mechanical recycling of multilayer films via forced assembly coextrusion. The originality of this study consists in limiting the number of constituents, reducing/controlling the thickness of the layers and avoiding the use of tie layers. The ultimate goal is to improve the manufacturing of new products from recycled multilayer materials by simplifying their recyclability. Within this framework, new structures were developed with two polymer systems: polyethylene/polypropylene and polyethylene/polystyrene, with nominal micro- and nanometric thicknesses. Hitherto, the effect of the multi-micro/nanolayer architecture as well as initial morphological and mechanical properties was evaluated. Several recycling processes were investigated, including steps such as: (i) grinding; (ii) monolayer cast film extrusion; or (iii) injection molding with or without an intermediate blending step by twin-screw extrusion. Subsequently, the induced morphological and mechanical properties were investigated depending on the recycling systems and the relationships between the chosen recycling processes or strategies, and structure and property control of the recycled systems was established accordingly. Based on the results obtained, a proof of concept was demonstrated with the eco-design of multi-micro/nanolayer films as a very promising solution for the industrial issues that arise with the valorization of recycled materials.

## 1. Introduction

Since the discovery of polyethylene and polypropylene during the 1950s, polymers have become integrated into all areas of our daily life. Nowadays, annual plastics production in Europe fluctuates around 60 million tonnes [1]. The packaging (39%) and building (19.7%) sectors are the biggest end-use markets for plastics, whereas the agricultural sector represents 3.3% of total plastic demand [1]. Among all plastic materials applications, flexible films have become very popular, mainly due to their versatility, lightness, resistance and printability. Currently, 17% of the world’s flexible film production consists of multilayer films [2]. These structures are obtained by coextrusion, which is an industrial process widely used to form films that are suitable for various products including food packaging, medical applications and, recently, microelectronics and nonlinear optics with several thousands of layers [3,4].

Multilayered polymer systems are produced to satisfy specific requirements for high value-added applications such as gas barrier films, robust mechanical systems and optical applications [5], and coextrusion is a process that combines multiple polymers. This is done via two or three extruders using a feedblock system in which melted polymers (from separate extruders) are brought together. Each component of the multilayer structure provides its own end-use characteristics. For example, low-density polyethylene (LDPE) and high-density polyethylene (HDPE) are the most common polymers used in the consumer packaging sector, followed by polyethylene terephthalate (PET), polypropylene (PP) and polystyrene (PS). For agricultural applications and other non-consumer packaging, LDPE and linear low-density polyethylene (LLDPE) are the most employed materials [6].

Nevertheless, the increasing generation and accumulation of non-biodegradable waste is becoming a high-profile public issue, since huge amounts of plastic packaging products are currently designed to have a short service life owing to their low cost and easy production [2]. In the European Union, around 25 million tonnes of post-consumer plastic waste are generated every year. An increase of the total volume of flexible consumer packaging is predicted from 27.4 (2017) to 33.5 million tonnes (2020) is predicted [2,6]. Most consumers have a negative perception of plastic packaging because of the considerable amount of waste produced in their daily lives. All things considered, it is of great importance to find new solutions for the valorization of multilayered films. Hence, a novel approach from eco-design to recycling of multilayer structures should be considered as an alternative if mechanical recycling can be assumed.

A design for recycling has been intensively promoted by the European Union in the last few years, in the context of the Circular Economy [7]. The strategy consists in developing new products so that they can be recycled at the end of their service life. The design of flexible polymer films has a large impact on both end-of-life (recyclability) and the degree to which they can incorporate recycled materials. Design for recycling is encouraged via the implementation of extended producer responsibility (EPR) schemes, in which the end-of-life cost is an economic motivation for the producers [8].

Recycling is a key factor to close the loop in the circular economy. However, the fact that multilayer films are made of different types of polymers causes their recycling to be even more challenging. Considering that complex blends are obtained after the recycling process, compatibilizers need to be added into the blends, but this process can create new issues that need to be further solved. The use of strategies such as structure simplification and eco-design are key factors in increasing the recyclability of the materials [2].

The starting point for the eco-design dynamic is the secondary raw material obtained from the recycled polymer waste that comes from a product that has reached the end of its service life. Nonetheless, as Ragaert et al. [8] have explained, design for recycling must consider multiple factors. This starts with an extensive characterization of the recycled polymers in order to identify their strengths and weaknesses, followed by finding potential new or existing products in which the recycled materials can be incorporated. Then, the design of the new products should be adapted for manufacturing using recycled materials. In addition, for some product requirements, cost-effective strategies can be applied in order to upgrade the quality of the recycled material. This can involve the addition of small amounts of additives such as compatibilizers or stabilizers.

Most European recycling companies work with two types of waste streams: polyethylene- and polyethylene terephthalate (PET)-based streams. However, the waste stream of PE-based films can be contaminated by the presence of other types of films containing polypropylene and polystyrene (PS). Considering the latter, for the present investigation, two polymer blend systems were studied and discussed: polyethylene (PE)/polypropylene (PP) and polyethylene (PE)/polystyrene (PS), since the combinations of these polymers are frequently found in the waste streams of recycling companies. Often, they are used together in the manufacturing of products and cannot be easily separated from each other. Upon waste reception, optical, ballistic densitometer and infrared sorting is applied. That said, most of the time, none of these automatic sorting procedures are efficient enough to completely separate polyethylene from polypropylene and polystyrene. Typical waste streams contain between 5 and 10 wt% polypropylene or polystyrene. Generally, a deterioration of the properties caused by the incompatibility between PE/PP and PE/PS is one of the major problems in processing mixed plastic wastes.

For PE/PP blends, numerous studies have been performed on the mechanical properties of mixed plastics, usually with a focus on neat materials. It has been found that the presence of PP in a PE matrix with a ratio between 5 and 30 wt% promotes an increase of the Young’s modulus and tensile strength at yield. However, a significant decrease of the elongation at break and the impact strength has also been observed, since polyethylene becomes rigid and more fragile upon breaking [9]. The PE/PP incompatibility has been proved mostly by microscopic and calorimetric analyses [10,11]. Since PE and PP are incompatible in the melt, the blend behaves as a two-phase mixture. The weak interfacial bond between both phases explains the poor mechanical properties, which are directly linked to the blend morphology. Therefore, since PE and PP are immiscible, a theoretical model for the properties of mixtures cannot be effectively applied [9].

Numerous efforts have been made to improve the compatibility between polyethylene and polypropylene. As found in the literature, the incorporation of a compatibilizer is the most successful technique for improving the morphology and phase dispersion by reducing the interfacial tension and enhancing the adhesion between polymer domains, which leads to an improvement of the mechanical properties. Kazemi et al. [12] summarized the most common graft or block copolymers used as compatibilizers, which include segments similar to the blend components. Styrenic block copolymers (SBC), ethylene-propylene elastomers (EPR), including ethylene-propylene-diene copolymer (EPDM), ethylene-vinyl acetate (EVA) and ethylene-octene copolymers (EOC) are the most commonly used in industry [12].

From a thermodynamic point of view, PE/PS systems are immiscible when blended. Indeed, blending such polymer pairs usually leads to materials with poor mechanical properties and a heterogeneous morphology. To improve the final properties of immiscible blends such as PE/PS without the presence of compatibilizers, multilayer coextrusion has been used to combine several systems with semi-crystalline polymers as confined materials against amorphous polymers with layer thicknesses ranging from 100 to 10 nm [13,14,15,16]. With the decrease of compatibilizers which seems to be complex in the classical multiphase polymer blends [17], forced assembly layer coextrusion seems to be a promised route to obtain micro-/nanostructured immiscible polymer systems. Owing the geometrical and macromolecular confinement and with the decrease of a layer’s thickness, the crystalline morphology of the system gradually evolves from a three-dimensional (3D) spherulite morphology into a one-dimensional (1D) lamellar morphology [15,16,18,19,20].

A systematic study of HDPE nanolayers sandwiched between thicker polystyrene (PS) in multilayer coextruded systems has been performed in previous investigations [16]. Interestingly, as the layer thickness of HDPE decreased from the micro- to the nanoscale, a significant reduction in crystallinity was observed from 60% to 33%. This result was associated with discoidal morphologies in the microscale (>100 nm) that transformed into long bundles of edge-on lamellae in HDPE nanolayers (<100 nm). Furthermore, the confined crystallization of polypropylene (PP) against a hard polymer (PS) with variable layer thicknesses and compositions in multilayer systems has been investigated [15]. The changes in layer thickness and composition of the component polymers strongly affected the mechanical properties and morphology of the multilayered film. When the film thickness decreased, however, the morphology passed from continuous and homogeneous to irregular, followed by a significant change in the mechanical properties, specifically the elongation at break [15].

The final objective of this investigation was to study the complex PE/PP and PE/PS systems with a new approach from design to recycling. For this purpose, a design of multi-micro/nanolayer films with a different number of layers is proposed, in order to improve the manufacturing of novel products using recycled materials and to simplify their own recyclability.

In the case of PE/PP compatibilization, multi-micro/nanolayer films were prepared by cast forced assembly coextrusion, in a process intended to vary the number of layers. A selected compatibilizer was incorporated into the central layer, in order to improve the adhesion between polyethylene and polypropylene. Next, the multilayer films were recycled with three different mechanical recycling processes. Finally, the influence of the number of layers and the recycling systems on the morphology and mechanical properties of the PE/PP films was evaluated.

Moreover, multi-micro/nanolayer films with polyethylene (PE) and polystyrene (PS) were also obtained by coextrusion, varying the number of layers. Following this, the structures were recycled with two different recycling processes, and the effect of the number of layers as well as the PE/PS compositions on the mechanical properties and morphology was studied.

## 2. Experimental Section

### 2.1. Materials and Sample Preparation Methodology

The properties of the materials used in this work are described in Table 1. To study the compatibilization between PE and PP, multilayer films with linear low-density polyethylene (LLDPE) and 10 wt% PP were first prepared. Then, in order to improve the adhesion between PE and PP, an ethylene-octene copolymer (EOC) was selected as a compatibilizer (Table 1) with a fixed rate of 7 wt%. As a second step, multilayer films composed of 50 wt% linear polyethylene (LDPE) and 50 wt% polystyrene (PS) were prepared in order to study the PS/PE compatibilization and the effect of confinement on crystallization.

### 2.2. Cast Forced Assembly Multilayer Coextrusion

**Figure 1:**
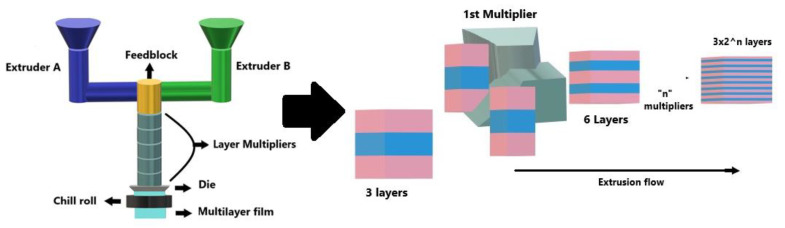
Schematic illustration of layer multiplication in the homemade multilayer coextrusion setup.

In this project, multilayer films were fabricated using a homemade multilayer coextrusion setup as displayed in Figure 1. From the feedblock, the three initial layers flowed through a sequence of layer multiplication dies. These dies divided the melt vertically, then spread it horizontally to the original width, and finally stacked the layered melt stream, while maintaining a constant melt thickness [4]. The final number of layers was determined as a function of the number of multiplication dies, which were placed in series between the feedblock and the final film or sheet exit dies (Figure 1). A set of “n” multipliers led to a film of 3 × 2^n^, since a three-layered feedblock was used for the PE/PP systems. For the PE/PS systems, however, the use of a two-layered feedblock and a set of “n” multipliers led to a film with 2 × 2^n^ layers. The temperature used during the coextrusion process was 230 °C for the extruders, multipliers and die. The chill roll was set to 40 °C for the PE/PP materials and to 60 °C for the PE/PS materials with a drawing speed of 1.27 m/min for both systems, in order to obtain a film thickness of 200 ± 50 µm. The feedblock configuration used for the PE/PP systems was B/A/B, whereas for PE/PS it was B/A, with “A” and “B” corresponding to the extruders displayed in Figure 1. It is important to mention that only symmetrical configurations were used in this work.

For the B/A/B feedblock configuration, two PE/PP systems were extruded: (a) PP/PE/PP, and (b) PP/PE + 7 wt% EOC/PP, with a volumetric flow of 90/10. Meanwhile, for the PE/PS systems, with the B/A feedblock configuration, films with volumetric flows of 50/50 and 10/90 were obtained, keeping the total thickness of the film constant around 200 μm.

All investigated multilayer films are listed in Table 2, where “n” is the number of multipliers and “N” the corresponding number of layers. For the PE/PP systems, the estimated nominal thickness for each layer with a B/A/B film configuration was calculated using Equations (1) and (2), in which “$\varphi_{A}$” and“$\varphi_{B}$” represent the volume fraction of A and B, respectively, “$h_{total}$” is the total film thickness and “*n*” is the number of multipliers:

(1) $$h_{nomA}=\varphi_{A}\frac{h_{total}}{2^{n}}$$

(2) $$h_{nomB}=\varphi_{B}\frac{h_{total}}{2\times 2^{n}}$$

However, for the PE/PS systems with a B/A configuration, the nominal layer thickness was calculated using only Equation (1), for both A and B components with different volume fractions (50/50 and 10/90 for A/B, respectively), with “$h_{total}$” as the total film thickness and “*n*” as the number of multipliers used.

### 2.3. Mechanical Recycling Processes

Three laboratory-scale mechanical recycling processes were performed for the multilayer films obtained by coextrusion. These recycling processes included different steps such as:

- Grinding: The multilayer films were ground with a blade grinder, leading to the production of small flakes.
- Cast-film extrusion: The flakes obtained from the grinding of multilayer films were used as raw material to feed the extruder. Monolayer films with a thickness of 200 µm were obtained as a result, with an extrusion temperature of 210 °C. The same extruder as the one shown in Figure 1 was used without the feedblock and multiplier elements, in order to obtain monolayer films.
- Extrusion (mini twin-screw extruder): Flakes obtained from the grinding of multilayer films were used as raw material to feed the extruder. The extrusion process was performed at 210 °C. Then, the obtained compounds were pelletized in a cutting machine. The extrusion process was performed using a HAAKE mini co-rotating twin-screw (mini CTW) machine (Thermo-Scientific, Waltham, MA, USA).
- Injection molding: Depending on the recycling process used, flakes (obtained by grinding) or pellets (prepared with the mini twin-screw extruder) were used as raw material to feed the injection-molding machine. A piston injection-molding HAAKE™-Mini-jet was used in order to obtain flat rectangular specimens for tensile testing. Sample dimensions of 2 × 4 mm^2^ (thickness × width) were obtained. The temperature of injection was 220 °C and the injection pressure was 550 bars for 8 s. Then a packing pressure of 450 bars was applied for 8 s.

The multi-micro/nanolayer films studied herein were recycled according to the following:

- R1→ Steps applied: Grinding + Monolayer cast film extrusion
- R2→ Steps applied: Grinding + Injection molding
- R3→ Steps applied: Grinding + Mini twin-screw extrusion+ Injection molding

### 2.4. Mechanical and Morphological Characterization

#### Tensile Testing

The tensile tests were performed with an electromechanical testing machine (INSTRON, Norwood, MA, USA). The specimens used had a rectangular shape with dimensions of 4 (±0.5) mm in width, and 50 (±0.1) mm in length. Each sample was clamped into the machine with one end held at a fixed position and the other end displaced at a constant rate of 50 mm/min, using a load cell of 100 N. Data was collected with a chart that monitored the force as a function of the displacement. However, the use of calibrated instrumentation to measure the changes in cross-sectional area during deformation was not accurate enough for thin films such as the multilayer materials in question as Li and al. from the Baer group described in their previous studies [18]. Hence, only engineering stress and strain data were analyzed in this study. The engineering stress of the samples was calculated using Equation (3) (in which “*F*” is the force causing a given deformation, and “*A*” is the area). Then, using Equation (4) we calculated the engineering strain, with “$l_{o}$” as the initial length and “Δl” the change in length. The elongation and tensile strength at break were determined from the stress-strain plot. Tests were performed only in the machine direction:

(3) $$\sigma_{eng}=\frac{F}{A}\;\;\left(\frac{N}{m^{2}}\right)$$

(4) $$\;\varepsilon_{eng}=\frac{\Delta l}{l_{o}}\times 100$$

### 2.5. Scanning Electron Microscopy

The influence of the recycling system on the morphology of the PE/PP and PE/PS multilayers was studied by scanning electron microscopy. For the PE/PP systems, the observations were performed with the specimens obtained after recycling processes R2 and R3. The samples were fractured in liquid nitrogen at a temperature below the glass transition temperature (Tg), and the observations were performed directly without further treatment on a QUANTA 250 FEG microscope (FEI, Hillsboro, OR, USA) in high-vacuum mode.

In the case of the PE/PS multilayer systems, the specimens were first stained by ruthenium tetroxide vapor (RuO_4_) for two days and then placed between two epoxy resin plates until consolidation was achieved. Then, the samples were cut and cross-sectioned perpendicularly to their surfaces but parallel to the extrusion direction using a cryo-ultramicrotome (EM UC7, LEICA, Wetzlar, Germany) at room temperature.

## 3. Results and Discussion

### 3.1. PE/PP Multilayer Systems

#### 3.1.1. Characterization of the Mechanical Properties

##### Effect of the Number of Layers

**Figure 2:**
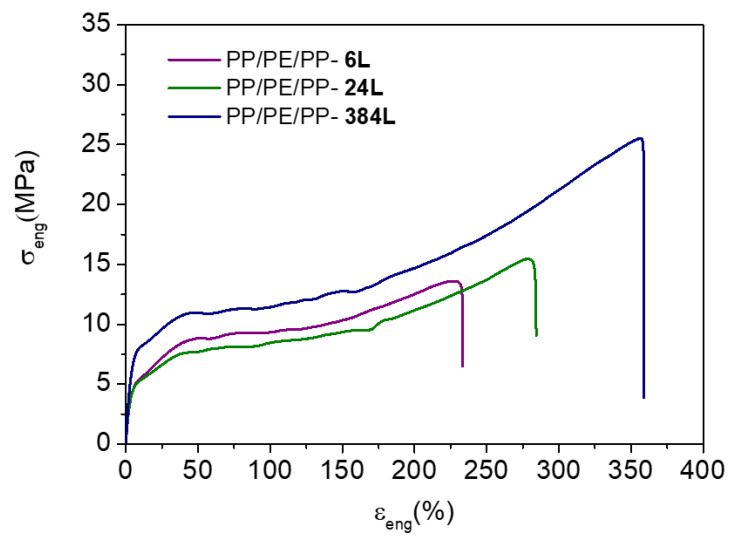
Engineering stress-strain plot of the PP/LLDPE/PP multilayer films with different numbers of layers in the machine direction (MD).

This section is devoted to the effect of the layer thicknesses on the tensile properties of PP/LLDPE/PP multilayer films with 6, 24 and 384 layers. It is important to remember that the PP/LLDPE/PP multilayer films were prepared with a 90/10 composition of LLDPE/PP. The nominal layer thicknesses of the PP/LLDPE/PP films for a B/A/B coextrusion configuration are displayed in Table 2. As can be observed in Figure 2 and Table 3, the number of layers greatly influenced the tensile properties: there was a direct relation to the thicknesses of the nominal layers. The PP/LLDPE/PP-6L and PP/LLDPE/PP-24L were microlayered films. The thicknesses of the nominal layers for PP/LLDPE/PP-6L film were 90 µm and 5 µm for the internal (LLDPE) and external layers (PP), respectively. The PP/LLDPE/PP-24L had nominal thicknesses of 24 µm and 1.3 µm for the internal and external layers, respectively. When the number of layers was increased, however, their nominal thicknesses were decreased, which led us to obtain a nanolayered film. This was the case for the PP/LLDPE/PP-384L films for which the external layer thickness was reduced down to 97 nm.

In the case of the microlayered films, PP/LLDPE/PP-6L and PP/LLDPEPE/PP-24L showed relatively comparable tensile strengths at yield and break. However, the elongation at break exhibited a slight increase with the number of layers due to the presence of thinner PP layers, which according to the literature exhibit superior behavior as compared with LLDPE [9]. It was thus observed that the nanolayered PP/LLDPE/PP-384L presented the highest tensile strength at yield and break (i.e., 8.3 MPa and 25.6 MPa) compared with the microlayered films, as well as the highest elongation at break. These results are quite interesting, since the nanolayered films exhibited a brittle and ductile behavior at the same time. The significant number of layers (384 L) and the thinner PP confined layers (97 nm) were the reasons behind the surprising mechanical response of PP/LLDPE/PP-384L.

Moreover, the tensile properties of the PP/LLDPE/PP multilayer films were compared with the monolayer multicomponent (PE/PP) films as described in Appendix A (Table A1). We observed that the microlayered films (PP/LLDPE/PP-6L and PP/LLDPE/PP-24L) presented tensile strength and elongation at break values closer to the monolayer PE/PP blend (Table A1). This reflects the poor interaction between the polyethylene and polypropylene, despite the microlayered configuration used. The nanolayered PP/LLDPE/PP-384L film, on the other hand, displayed a greater tensile strength and elongation at break than the compatibilized PE/PP monolayer film (with 7 wt% EOC), much closer to the tensile properties of the pure PE-based film (Table A1).

These results are very promising, since they confirm the importance of the design for recycling concept, as well as the number of confined layers, for improving physical compatibilization. Choosing a suitable configuration of layer thickness and composition makes it possible to achieve the desired mechanical properties without using compatibilizers.

##### Effect of a Compatibilizer

**Figure 3:**
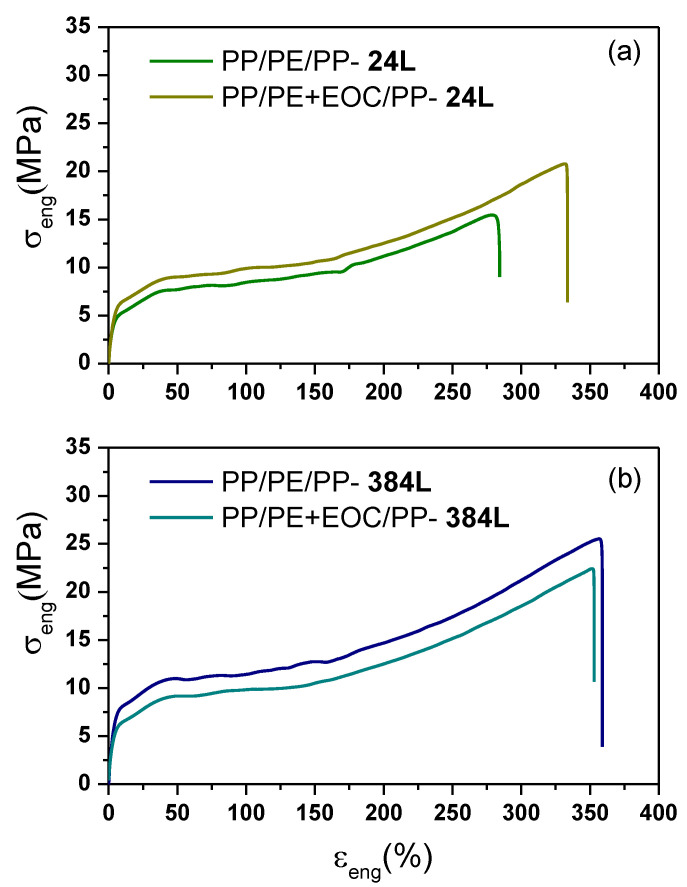
Engineering stress-strain plot of the PP/LLDPE/PP and PP/LLDPE + EOC/PP multilayer films (MD). Influence of the EOC compatibilizer on the films with: (**a**) 24 layers and (**b**) 384 layers.

The influence of an EOC compatibilizer on the mechanical properties of the PP/LLDPE/PP multilayer films is discussed in this section. The tensile properties of PP/LLDPE + EOC/PP-24L and PP/LLDPE + EOC/PP-384L are displayed in Figure 3 and Table 3. Considering the case of the microlayered films, the PP/LLDPE + EOC/PP-24L presented higher elongation at break (331.5 ± 18%) than the uncompatibilized PP/LLDPE/PP-24L film (285 ± 27%). This result was predictable considering the elastomeric nature of the EOC and the influence of this compatibilizer in enhancing the adhesion between PE and PP (Figure A2, Appendix A). However, the slight increase in tensile strength with the presence of EOC was interesting and required further investigation.

In the case of the nanolayered films with 384 layers, the effect of the EOC on the elongation at break was almost unnoticeable, compared with the microlayered PP/LLDPE/PP-24L and PP/LLDPE + EOC/PP-24L films. However, a decrease of the tensile strength at yield and break was observed, due to the elastic nature of the EOC. Additionally, we observed that the compatibilized PP/LLDPE + EOC/PP-24L showed similar tensile properties to the PP/LLDPE/PP-384L system without compatibilizer (Table 3).

These results confirmed our previous statement regarding the design and appropriate selection of the multilayer configuration. With multi-nanolayer films, it was possible to enhance the mechanical properties between incompatible polymers such as PE/PP without using compatibilizers. This would give rise to savings in the industrial manufacturing of recycled blends since compatibilizers represent an additional cost.

##### Influence of the Recycling Process System

**Figure 4:**
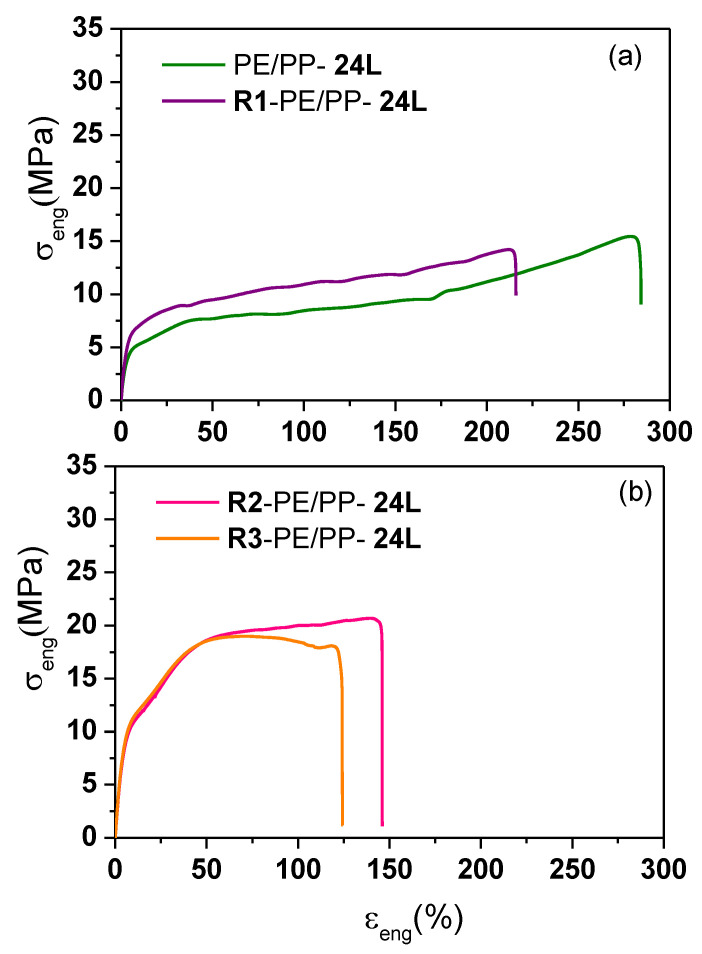
Engineering stress-strain plot of the recycled PP/LLDPE/PP-24L multilayer films (MD) obtained from different recycling processes: (**a**) R1 and (**b**) R2 and R3.

The tensile properties of the PP/LLDPE/PP-24L neat multilayer film were compared with those of its recycled monolayer version obtained from the recycling process R1 described in the Experimental Section 2.3. This mechanical recycling process involved grinding and extrusion steps, after which the PP/LLDPE/PP neat film layers were fractured. As can be seen in Figure 4a and Table 4, the elongation at break of the PP/LLDPE/PP-24L neat film (285 ± 27%) decreased by 25% after the recycling process (214 ± 20%), which was expected considering the degradation of the film during the recycling.

For this reason, studies were performed on the specimens obtained from the recycling processes R2 and R3 of PP/LLDPE/PP-24L. The difference between recycling processes R2 and R3 is the additional extrusion step between the grinding and the injection molding. As displayed in Figure 4b, the shape of the stress vs. strain plot was very different from the others since these latter (R2 and R3) specimens had been obtained from injection molding. Consequently, these latter specimens could not be directly compared with monolayer films. Considering the effect of the type of recycling system, we found that the tensile strength at yield and at break was practically the same. However, the elongation at break of R3-PP/LLDPE/PP-24L decreased by 15% (120 ± 11%) compared with R2-PP/LLDPE/PP-24L (141 ± 14%). This was probably due to the degradation that theR3-PP/LLDPE/PP-24L film suffered during the additional extrusion step before the injection molding. These results highlight the impact of the recycling process on the mechanical properties of the multilayer films.

#### 3.1.2. Morphological Characterization of the Recycled Multilayer Films

**Figure 5:**
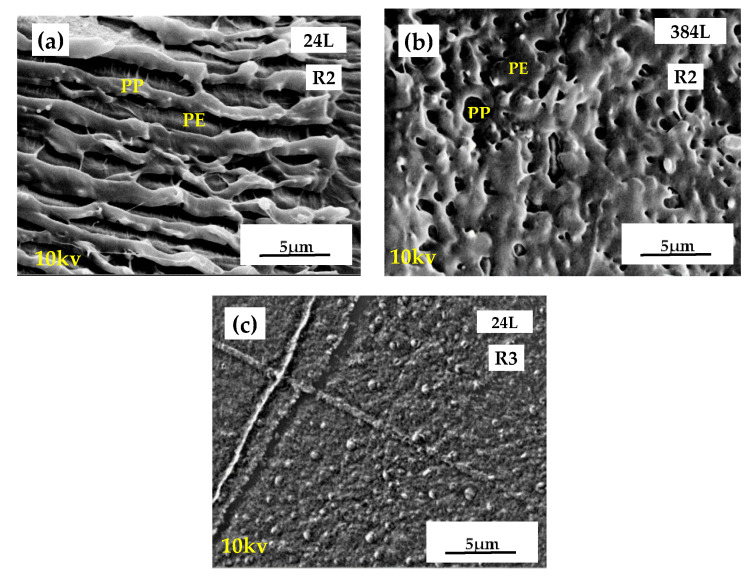
SEM micrographs of (**a**) the PP/LLDPE/PP-24L and (**b**) the PP/LLDPE/PP-384L films recycled with the R2 recycling process, and (**c**) the PP/LLDPE/PP-24L film recycled with R3.

The PP/LLDPE/PP-24L and PP/LLDPE/PP-384L films were selected to evaluate the effect of the number of layers and the nominal thickness on the PP/LLDPE/PP film morphology after recycling process R2. As can be observed in Figure 5a, the microlayered recycled film PP/LLDPE/PP-24L maintained its homogeneous multilayered morphology even after recycling. The different PE/PP layers were easily observable, as indicated in Figure 5a, where the smoother surface was believed to correspond to the PE phase.

**Figure A1:**
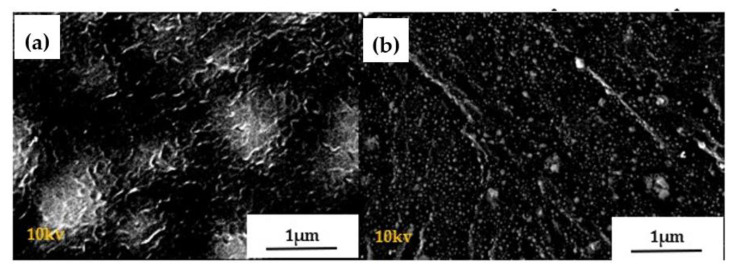
High-vacuum SEM images of PE/PP monolayer films: (**a**) PE/PP (90/10) (without compatibilizer) and (**b**) PE/PP/EOC (83/10/7).

**Figure A2:**
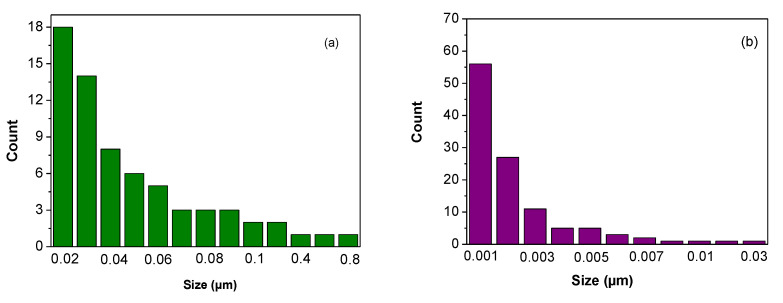
Size distribution of the PP droplets in the PE matrix: (**a**) PE/PP (without compatibilizer) and (**b**) PE/PP/EOC (83/10/7).

As for the morphology of the nanolayered PP/LLDPE/PP-384L recycled film, the layers were no longer visible after the recycling process (Figure 5b). Since PP/LLDPE/PP-384L had a nominal layer thickness at the nanometric scale, the grinding stage was sufficient to completely destroy the layered morphology. These observations were very interesting, because as seen in Figure 5b, R2-PP/LLDPE/PP-24L exhibited a dispersed morphology, as presented by PE/PP monolayer blends in Appendix A (Figure A1).

Next, the influence of the mechanical recycling system was evaluated for the PP/LLDPE/PP-24L recycled films. As observed in Figure 5c, the effect of the extrusion step carried out after the grinding and before the injection molding greatly affected the morphology, cf. Figure 5a. The extrusion step of recycling process R3 significantly fractured the microlayered structure of PP/LLDPE/PP-24L. A dispersed morphology was thus obtained, similar to the dispersed morphology observed with PE/PP monolayer films (Figure A1). These observations highlight the interesting microstructure of the blends created by the twin-screw extrusion. All these results confirm our previous statement regarding the impact of the recycling process on the mechanical properties and the morphology of the multilayered films.

### 3.2. PE/PS Multilayer Systems

#### 3.2.1. Study of the Mechanical Properties

##### Effect of the Number of Layers and Composition

**Figure A3:**
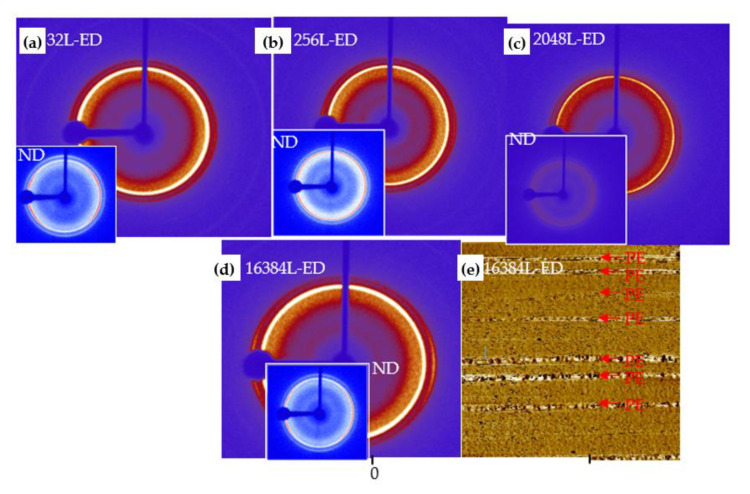
2D-WAXS profiles recorded with an X-ray beam in normal and parallel directions to the film plane for LDPE/PS multilayers: (**a**) 32L, (**b**) 256L, (**c**) 2048L, (**d**) 16380L and (**e**) example of an AFM image for a sample with 16380 layers.

**Figure A4:**
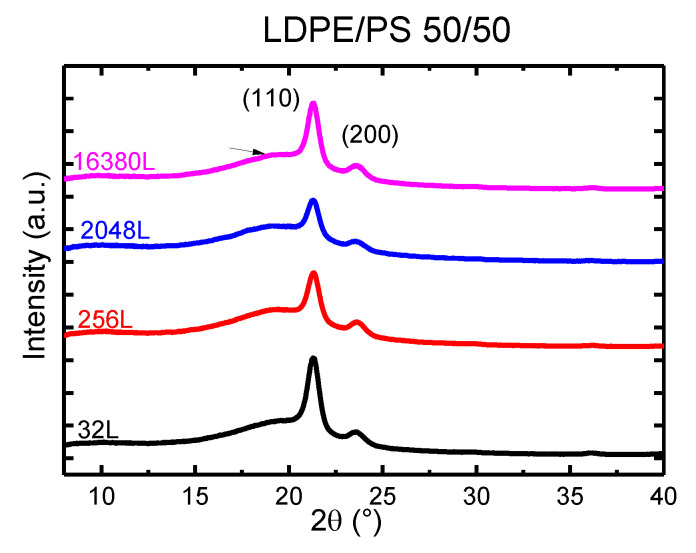
1D-WAXS profiles for LDPE/PS multilayers with different numbers of layers: 32L, 256L, 2048L and 16380L. The intensity was normalized with the thickness of the studied films. The scattering angle was denoted 2θ.

Films of LDPE/PS with 50/50 and 10/90 compositions and different numbers of layers (from 32 to 16,380 layers), as shown in Table 2, were studied with regard to the effect of the layer thickness and the composition on the mechanical properties. For these PE/PS multilayer systems, the thickness of each component and the number of layers was varied from the micro- to the nanometric scale, with a nominal thickness of around 100 nm for the 50/50 composition. This is very close to the thickness of lamellae. For the clarity purpose of the present paper, the effect of layer numbers and LDPE geometrical confinement on the crystalline properties are detailed in Appendix B and Appendix C. Both 2D WAXS and 1D-WAXS profiles are detailed in Appendix B in Figure A3 and Figure A4. The obtained results corroborate those obtained by DSC as described in Table A2, Appendix C.

**Figure 6:**
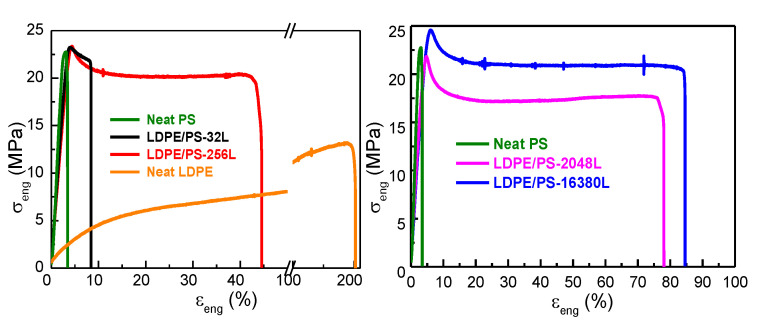
Engineering stress-strain plot of LDPE/PS multilayer 50/50 (wt/wt) films with different numbers of layers in the machine direction.

The stress-strain curves of multilayer LDPE/PS 50/50 films are displayed in Figure 6. We can see that the tensile strength at yield of the multi-micro/nanolayer structures was similar to that of PS and remained constant over 22MPa. Nevertheless, the number of layers had an influence on the elongation at break of the multilayered films, as shown in Table 5 and Figure 6. For example, the LDPE/PS-32L structure, with a nominal thickness of 6250 nm, exhibited an intermediate behavior between those of LDPE and PS. LDPE/PS-32L was found to be brittle and comparable to neat PS. However, for LDPE/PS-16380L, with a nominal thickness of 97 nm, the mechanical response of the multilayer film showed a higher ductility, which indicates that the brittle behavior observed with LDPE/PS-32L was due to the poor adhesion between the LDPE/PS interfaces at the microscale. Hence, as we increased the number of layers, the adhesion at the LDPE/PS interface improved due to the geometric confinement of the layers, which explains the increased ductility of the film. These results agree with previous studies of multilayered immiscible PP/PS films with varying compositions (from 90/10 to 10/90) reported by Scholtyssek et al., 2010 [15], who found that the elongation at break increased significantly with the decrease of nominal layer thickness at constant composition.

**Figure 7:**
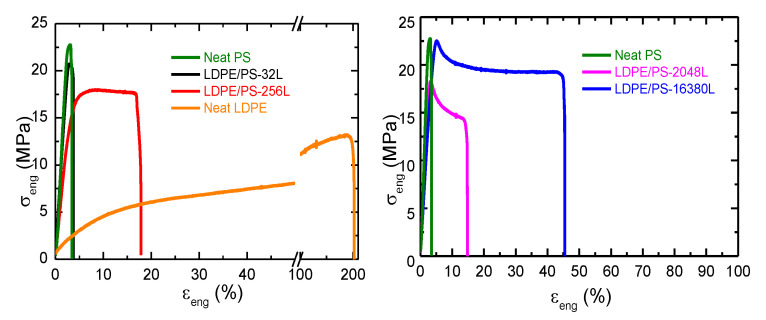
Engineering stress-strain plot of multilayered LDPE/PS 10/90 films (in the machine direction) with different numbers of layers.

For the multilayered structures with a 90/10 composition, the samples showed an intermediate behavior between the neat LDPE and PS, as seen in Figure 7 and Table 6. However, for the LDPE/PS-16380L film with a nominal layer thickness of ~95 nm, we observed an increase of the tensile strength at yield as well as at break. This can be explained by the effect of geometric confinement of LDPE crystals against the amorphous PS and also by the effect of on-edge orientation during the coextrusion process, as explained in detail in Appendix B and Appendix C.

Regarding the effect of the LDPE/PS composition, clear differences were found between the LDPE/PS films with 50/50 and 10/90 compositions, as shown in Figure 6 and Figure 7. The LDPE/PS 50/50 sample showed a higher elongation at break regardless of the number of layers, compared with LDPE/PS 10/90. These results demonstrate that the film’s ductility was not only influenced by the nominal layer thickness but also by the composition. A similar conclusion was put forward by Scholtyssek et al. [15], who stated that the composition has a significant effect on the ductility of multilayered films.

##### Influence of the Recycling Process on the Mechanical Properties

**Figure 8:**
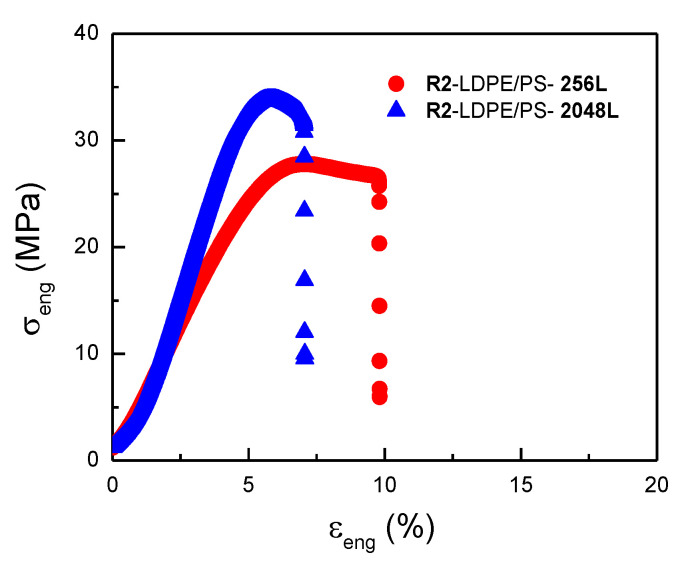
Engineering stress-strain plot of the recycled R2-LDPE/PS (10/90) multilayer films (MD) with different numbers of layers.

Next, we analyzed the effect of the different recycling processes (described in the experimental section) on the mechanical properties of the multilayer films. Figure 8 displays the effect of the number of layers on the recycled films denoted R2-LDPE/PS (10/90). A significant reduction of the elongation at break was seen for the recycled multilayer film with 2048 layers compared with its 256-layer counterpart. This was expected, since the nominal thickness of the LDPE/PS-2048L (10/90) film was thinner (~100 nm) than the multilayer film with 256 layers (~1 µm), thus reflecting the fragility of the layers during the grinding and injection steps of recycling process R2. Nevertheless, in terms of tensile strength at yield and at break, we observed the opposite effect with the number of layers. This can be explained by a geometric confinement effect of LDPE crystals against the amorphous PS, even after the R2 recycling process.

**Figure 9:**
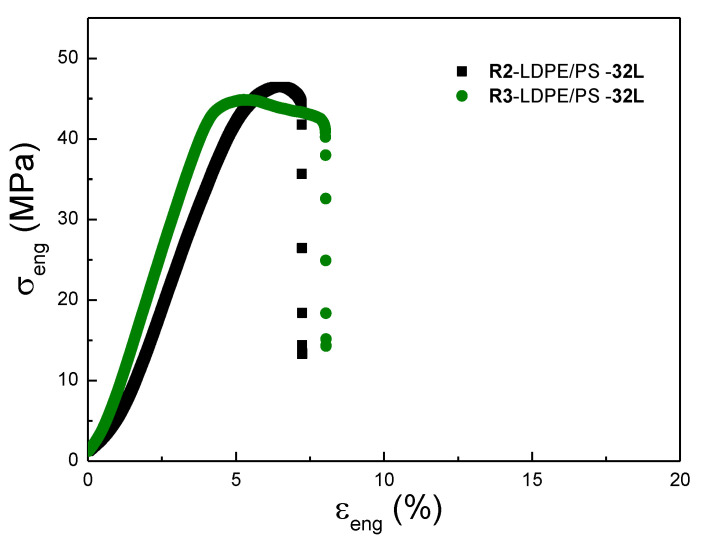
Engineering stress-strain plot of the recycled LDPE/PS (10/90)-32L multilayer films (MD) with the R2 and R3 recycling systems.

Next, Figure 9 compares the effect of recycling processes R2 and R3 on the mechanical properties of the LPDE/PS (90/10) with 32 layers. As a reminder, the difference between recycling processes R2 and R3 is the additional step of twin-screw extrusion between the grinding and injection molding steps. As displayed in Figure 9, both recycled films showed equivalent strengths at yield and at break, and the same was true for the elongation at break. These results indicate that the additional extrusion step for the R3 recycling process did not degrade the multilayer films as much as for the PE/PP systems (Figure 5). This can be due to the brittle behavior of the PS as compared with PE and PP.

#### 3.2.2. Morphological Characterization of the Recycled and Neat LDPE/PS Films

**Figure 10:**
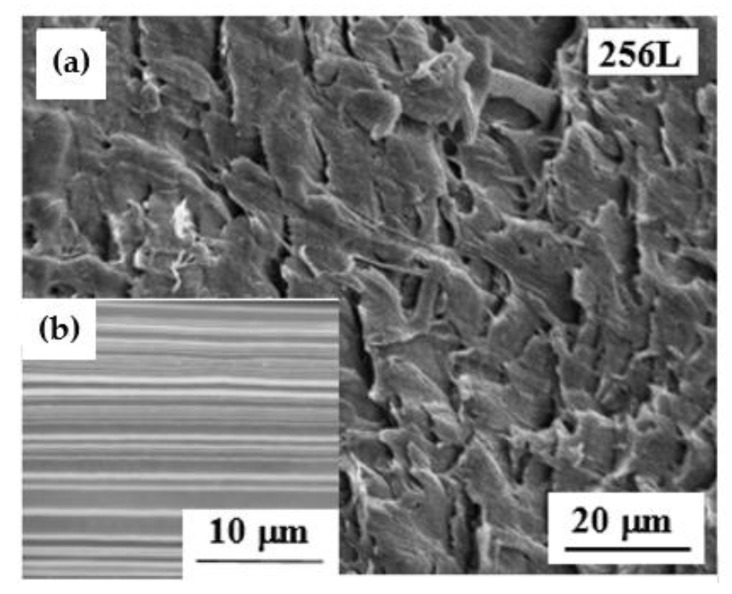
SEM micrographs of (**a**) the recycled R2-LDPE/PS-256L multilayer film and (**b**) the neat LDPE/PS-256L multilayer film with 50/50 composition.

SEM micrographs of neat and recycled LDPE/PS-256L with a 50/50 composition are displayed in Figure 10. These structures were analyzed over a cross-section in the machine direction in the aim of studying the effect of the recycling system on the morphology of the multilayered structure.

As shown in Figure 10b, the neat LDPE/PS-256L presented a continuous and regularly structured morphology. It is important to mention that the continuity as well as the uniformity of the multilayered films was greatly affected by the film thickness and composition, which is in agreement with the results obtained by Scholtyssek et al. [15]. However, with the recycled R1-LDPE/PS-256L film (Figure 10b) we could no longer differentiate between the layers. This proved that the grinding and extrusion steps were sufficient for destroying the layered morphology presented by the neat LDPE/PS-256L.

## 4. Conclusions

This paper presents a new approach to design for recycling. In order to improve the manufacturing of new products using recycled materials and to simplify their recyclability, we proposed a design of multi-micro/nanolayer films with different numbers of layers.

Considering that combinations of polyethylene/polypropylene and polyethylene/polystyrene are frequently found in recycling companies’ waste streams, these two polymer blend systems were analyzed and discussed in the present investigation. Generally, the deterioration of mechanical properties caused by an incompatibility between polyethylene/polypropylene and polyethylene/polystyrene is one of the major problems in processing mixed plastic wastes.

In order to study the compatibilization of polyethylene/polypropylene, PE/PP multi-micro/nanolayer films were prepared by coextrusion on a laboratory scale, by varying the number of layers. The EOC compatibilizer was incorporated in the central layer, so as to improve the adhesion between the PE/PP layers. Then, the multilayer films were recycled with three different mechanical recycling systems. With the results obtained from the mechanical characterization, we demonstrated that choosing the most suitable configuration for the multilayer films is a valuable way to boost the mechanical properties of the incompatible PE/PP systems. Indeed, with a multi-nanolayer film configuration, it was possible to enhance the mechanical properties between PE/PP without using compatibilizers. Moreover, a comparison between the various recycling systems demonstrated the impact of the recycling process on the mechanical properties of the multilayer films.

Next, a characterization of the morphology carried out with SEM showed that the nanolayered recycled films (from recycling process R2) presented a dispersed morphology with polyethylene as the continuous matrix. However, the microlayered recycled films (from recycling process R2) kept their layered morphology even after recycling. Meanwhile, the additional extrusion stage carried out prior to injection molding, in recycling process R3, greatly fractured the layered structure of the microlayered film. A dispersed morphology was observed when passing from micro- to nanolayer systems. All these results confirm our statement regarding the impact of the recycling process on the mechanical properties and the morphology of the multilayer films.

Moreover, structures with alternating layers of low-density polyethylene (LDPE) and polystyrene (PS) were also prepared by coextrusion of a varying number of layers and compositions. For the symmetrical composition (50/50), the multilayered films presented a brittle behavior with a nominal thickness at the microscale. However, as we decreased the nominal layer thickness down to the nanoscale, the multilayer films became more ductile, as the elongation at break was increased. This can be explained by the geometrical confinement and the orientation of LDPE chains during the coextrusion process. Additionally, the same trend was found for the asymmetrical composition (10/90), suggesting that the ductility of the films was not only influenced by the nominal thickness but also by the composition of the blends.

A continuous and regularly structured morphology of the neat multilayer films was observed with SEM analysis. However, after the R2 recycling process, the layered structure could no longer be observed, indicating that only the grinding and injection molding steps were sufficient to destroy the layered structure presented by the neat multilayer film.

Regardless of the polymer system studied, we have demonstrated that the design of multi-micro/nanolayer films is a very promising solution to the industrial issues that accompany the valorization of recycled materials, without the use of compatibilizers. It is, however, important to mention that the present study was performed exclusively at the laboratory scale as a proof of concept for further scale-up investigations. Moreover, a deeper understanding of the nanostructure mechanisms and morphological involved changes during recycling of the present systems will require further investigation in the future.

## Figures and Tables

**Table 1:** Summary of the polymers used for the PE/PP and PE/PS systems.

Polymer	LLDPE	PP	EOC	LDPE	PS
Manufacturer	Chevron Phillips	LyondellBasell	Dow Chemical	ExxonMobil 165	Crystal 637
Monomer type	Ethylene/1-Hexene Copolymer	Propylene/Ethylene Copolymer	Ethylene/Octene Copolymer		
Density (g/cm^3^)	0.918	0.900	0.875	0.922	1.05
MFI (g/10 min)	1.0 ^a^	0.85 ^b^	1.0 ^a^	0.33 ^a^	4 ^c^

^a^ 190 °C/2.16 kg; ^b^ 230 °C/2.16 kg; ^c^ 200 °C/5 kg.

**Table 2:** Characteristics of the PE/PP and PE/PS multilayer films obtained by coextrusion.

Films	Volumetric Flow (*v*/*v*)	Number of Multipliers (n)	Number of Layers (N)	Total Thickness(µm)	Nominal Layer Thickness (nm) B/A/B Configuration	
					A	B
	PE/PP systems					
PP/LLDPE/PP	90/10	1	6	200	50,000	25000
		3	24	210	23,625	1313
		7	384	250	1758	98
PP/LLDPE + EOC/PP		3	24	210	23,625	1313
		7	384	250	1758	98
**Films**	**Volumetric Flow (*v*/*v*)**	**Number of Multipliers (n)**	**Number of Layers (N)**	**Total Thickness** **(µm)**	**Nominal Layer Thickness (nm)** **B/A Configuration**	
					**A**	**B**
PE/PS systems						
LDPE/PS (10/90)	10/90	4	32	200	1250	11,250
		7	256		156	1406
		10	2048		20	176
		13	16,384		3	22
LDPE/PS (50/50)	50/50	4	32		6250	6250
		7	256		781	781
		10	2048		97	97
		13	16,384		12	12

**Table 3:** Tensile strength properties in the machine direction (MD) of the PP/LLDPE/PP and PP/LLDPE + EOC/PP multilayer films with different numbers of layers.

Multilayer Films	Tensile Strength at Yield (MPa)	Tensile Strength at Break (MPa)	Elongation at Break (%)
PP/LLDPE/PP-6L	5.5 ± 1.7	13.5 ± 3.3	234.7 ± 20.0
PP/LLDPE/PP-24L	4.5 ± 0.5	15.5 ± 1.3	284.5 ± 27.4
PP/LLDPE/PP-384L	8.3 ± 1.2	25.6 ± 4.0	355.5 ± 28.0
PP/LLDPE + EOC/PP-24L	4.3 ± 1.0	20.6 ± 2.5	331.5 ± 18.1
PP/LLDPE + EOC/PP-384L	5.9 ± 1.6	22.4 ± 2.5	351.1 ± 23.0

**Table 4:** Ensile strength properties in the machine direction of the recycled PP/LLDPE/PP-24L multilayer films following different recycling processes.

Recycled Samples	Tensile Strength at Yield (MPa)	Tensile Strength at Break (MPa)	Elongation at Break (%)
Monolayer film			
R1-PP/LLDPE/PP-24L	6.3 ± 1.3	15.5 ± 3.5	213.7 ± 20.0
Tensile test rectangular specimen			
R2-PP/LLDPE/PP-24L	10.0 ± 0.5	20.3 ± 0.2	140.5 ± 14.2
R3-PP/LLDPE/PP-24L	10.8 ± 0.3	18.1 ± 0.1	120.3 ± 11.0

**Table 5:** Mechanical properties of the LDPE/PS 50/50 (wt/wt) films with different numbers of layers.

Films	Tensile Strength at Yield (MPa)	Tensile Strength at Break (MPa)	Elongation at Break (%)
LDPE/PS-32L	22 ± 2.8	22 ± 2.2	8 ± 2.4
LDPE/PS-256L	22 ± 6	20 ± 2	45 ± 18
LDPE/PS-2048L	22 ± 2.6	18 ± 2.6	78 ± 27
LDPE/PS-16380L	24 ± 2.2	20 ± 2	84 ± 20

**Table 6:** Mechanical properties of the LDPE/PS 10/90 (wt/wt) films with different numbers of layers.

Films	Tensile Strength at Yield (MPa)	Tensile Strength at Break (MPa)	Elongation at Break (%)
LDPE/PS-32L	20 ± 3.2	20 ± 3	4 ± 0.5
LDPE/PS-256L	18 ± 0.5	17.5 ± 1	16 ± 14
LDPE/PS-2048L	18 ± 1.1	14 ± 1.5	14 ± 3
LDPE/PS-16380L	22 ± 2.6	19 ± 2	45 ± 14

## Data Availability

Not applicable.

## Appendix A. Mechanical Properties and Morphological Characterization of PE/PP Monolayer Films

Model PE/PP monolayer films were prepared by extrusion in order to study the compatibilization between PE and PP with simpler structures. Model blends were prepared with neat LLDPE and PP selected for this study (Table 1) as well as the compatibilizer EOC, in order to improve the adhesion between PE and PP.

Monolayer PE/PP films with and without compatibilizer EOC were obtained by blown film extrusion using a co-rotating twin-screw extruder with an annular blow die with a 50-mm diameter coupled to a melt pump. The temperature profile during extrusion was gradually increased from 190 °C to 220 °C from the feeding, tothe compression, to the pumping zone. The molten polymer in the form of a tube exited from the die at 220 °C and was then drawn upward by the take-up device. When the process started up, air was introduced at the bottom of the die to inflate the tube in order to form a bubble. The tube was then flattened in the nip rolls and taken up by the winder. An air ring was also used to rapidly cool the hot bubble and solidify it above the die exit. The film dimensions were determined by the blow-up ratio (BUR) and the take-up ratio (TUR). BUR is defined as the ratio between the final bubble diameter (Df) and the die diameter (D0), which can be controlled by varying the air pressure. TUR, on the other hand, is defined as the ratio between the speed of the take-up device (Vf) and the extruded material velocity (V0) at the die exit. For the present study, a drawing speed of 3.7 m/min with a fixed BUR and TUR was used in order to obtain films with similar thicknesses of around 30 ± 5 µm. All the films were prepared with the same extrusion parameters.

### Appendix A.1. Tensile Properties

Tensile testing of the monolayer films was performed under the same conditions and with the same parameters as explained in the experimental section. The samples had a rectangular shape, with dimensions of 4 (±0.5) mm in width, and 50 (±0.1) mm in length (ISO 527-1).

As observed in Table A1, the presence of PP in the PE/PP (90/10) film increased the tensile stress at yield, compared with the properties of the 100% PE film. However, it was also found that inclusion of PP in the PE continuous phase of the PE/PP (90/10) film led to lower deformability or elongation at break and tensile stress at break. The elongation at break of the 100%PE film (562.6 ± 30%) decreased by 59% compared with the PE/PP (90/10) film (228.1 ± 35%). As previously reported in numerous studies [17,18], blends of polyethylene and polypropylene are incompatible, resulting in poor mechanical properties. Next, we observed that the inclusion of the EOC compatibilizer reduced the tensile stress at yield and at break of the PE/PP (90/10) film. This was expected, due to the elastomeric nature of the EOC (Table 1). Conversely, the PE/PP (90/10) elongation at break increased by 5% with the addition of 7 wt% EOC. These observations confirm that EOC was a suitable candidate for enhancing the compatibility of our PE/PP blends.

**Table A1:** Tensile strength properties of the PE/PP monolayer films in the machine direction (MD).

Films (wt/wt)	Tensile Strength at Yield (MPa)	Tensile Strength at Break (MPa)	Elongation at Break (%)
100% PE	6.7 ± 0.6	44.3 ± 6	562.6 ± 30
90% PE 10% PP	8.2 ± 0.5	19.5 ± 2	228.1 ± 35
83%PE 10%PP 7% EOC	2.19 ± 0.2	16.4 ± 4	240.0 ± 48

### Appendix A.2. Morphological Characterization with SEM

The morphology of the PE/PP monolayer films was studied using scanning electron microscopy (SEM). The samples shown in Figure A1 were prepared with the aid of a cryoultramicrotome and were chemically stained with ruthenium tetroxide (RuO_4_) vapor for 2 h, in order to enhance the contrast of the samples’ different phases.

As can be seen in Figure A1a, the PE and PP phases were clearly visible. The continuous phase (black) corresponded to the polyethylene, while the dispersed phase (gray) represented the PP domains, which exhibited more brittle behavior compared with the polyethylene [9]. The SEM micrograph in (Figure A1a shows the phase morphology of the PE/PP monolayer film as a dispersion of PP droplets in the PE matrix. Figure A1b displays the morphology of the PE/PP with 7 wt% EOC as a compatibilizer. As can be observed, the addition of EOC promoted finer morphologies. Figure A2 reveals the PP droplet size distributions of the PE/PP films with and without compatibilizer. The addition of 7 wt% EOC into the PE/PP blend was sufficient to reduce the domain sizes, resulting in a finer morphology. The uncompatibilized PE/PP blend presented an average PP droplet size of 0.1 µm, whereas the EOC-compatibilized blend had an average PP droplet size of 0.01 µm. The compatibilizer resided at the interface between PE and PP and reduced their interfacial tension [12]. These observations confirm the improvement of some of the tensile properties of the PE/PP monolayer films by the presence of 7 wt% EOC as a compatibilizer.

## Appendix B. LDPE/PS Systems: Effect of the Number of Layers on the Crystalline Orientation of LDPE

The orientation and crystalline structure of LDPE in multilayered LDPE/PS films were characterized by 2D-WAXS patterns with an X-ray incident beam in the extruded and normal direction to the film plane, as seen in Figure A3. The LDPE/PS films exhibited two clear diffraction rings at 2θ = 21.33° and 23.66°, corresponding to (110) and (200) from the orthorhombic crystal structure of polyethylene superposed with the wide amorphous halo of PS (2θ = 18.9°), as seen from the 1-D profile for all multilayered structures in Figure A4. WAXS images of both normal and extruded directions of these multilayered structures revealed a slight equatorial concentration of the (200) planes and became more intense as we increased the number of layers, especially in the case of 16380L. This was an indication that the chains had aligned parallel to the film plane resulting in an on-edge orientation, in which the confined spherulite changed from three dimensions (3-D) to a compressed two-dimensional spherulite. This is mentioned in the literature as discoids nucleating at the LDPE/PS interface. The confinement effect of LDPE chains against amorphous PS in the normal direction(ND) gave rise to an on-edge orientation, in which the LDPE crystals were set parallel to the film plane, whereas in the extruded direction the on-edge oriented chains were stretched and extended along the equator during the coextrusion process.

## Appendix C. Thermal Characterization of the LDPE/PS Systems with Dynamic Scanning Calorimetry (DSC)

The DSC parameters for neat LDPE, multilayered films, and their blends with weight fractions of 50/50 are summarized in Table A2. The melting temperature remained relatively constant for all multilayered samples and lay between the melting temperature of neat LDPE and that of the blend. For the melting enthalpy of LDPE, a slight increase was observed, ranging from 24.5 J/g for 32L to 31 for 2048L, corresponding to the nominal thickness of 6250 nm and 195 nm, respectively. However, with a nominal layer thickness of 95 nm for 16380L, the melting enthalpy decreased down to 26 J/g, which was similar to the enthalpy obtained for the blend.

Indeed, a similar trend was observed for the crystallinity of the multilayered films, which increased from 8.5% for 32L to 10.5% for 2048L, but then decreased to 9% for the film with 16380 layers. This was very similar to the crystallinity of the blend, which meant that films with more than 2048 layers presented a crystallinity similar to that of a blend.

**Table A2:** DSC parameters for the LDPE/PS multilayers, the blend (50/50) and the neat polymer.

Sample	LDPE/PS—50/50				
	T_m_ (°C)	ΔHm (J/g)	Xc, DSC (%)	h_N_	L (nm)
LDPE	112	117	40	-	9.47
32	110.5	24.5	8.5	6250 nm	9.01
256	111.7	29.7	10	781 nm	9.38
2048	110.6	31	10.5	195 nm	9.04
16380	110	26.5	9	97 nm	8.81
Blend	113	25	8.5	-	9.81

An example of the morphology obtained by AFM for a sample with 16380 layers is presented in Figure A3. As shown, the LDPE nanolayers had a bright color, while the PS appeared as dark layers. These observations showed a morphology with continuous layers of PE and PS, with a large size distribution in the LDPE thickness, where the amorphous PS layers confined the 3-D spherulite of LDPE and were forced to bend and create two-dimensional discoids. The thickness of LDPE measured from the AFM micrographs was higher than the estimated value calculated from Equation (1).
